# Supplementary material for: PRAMEY: A Bovid-Specific Y-Chromosome Multicopy Gene Is Highly Related to Postnatal Testicular Growth in Hu Sheep
Source: Animals (Basel). 2022 Sep 12;12(18):2380. doi: 10.3390/ani12182380 (PMC9495132; doi:10.3390/ani12182380)
Supplement: Supplementary file 1 [file animals-12-02380-s001.zip › animals-1863621-supplementary.pdf]

PRAMEY: A Bovid-Specific Y-Chromosome Multicopy Gene Is Highly Related to Postnatal Testicular Growth in Hu Sheep

Shengwei Pei [1], Fang Qin [2], Li Wang [1], Wanhong Li [1], Fadi Li [1] and Xiangpeng Yue [1,*]

[1] Key Laboratory of Grassland Livestock Industry Innovation, Ministry of Agriculture and Rural Affairs; Engineering Research Center of Grassland Industry, Ministry of Education; State Key Laboratory of Grassland Agro-ecosystems; College of Pastoral Agriculture Science and Technology, Lanzhou University, Lanzhou 730020, China

[2] School of Pharmacy, Lanzhou University, Lanzhou 730000, China

* Correspondence: lexp@lzu.edu.cn

Supplementary Material

Supplementary Tables and Figures

Supplementary Tables

Supplement Table S1. The pairwise comparisons of the median copy number of *PRAMEY* among eight sheep breeds

	DP	EF	HS	SK	SMM	TS	TL	WSK
DP	–							
EF	nsd	–						
HS	**	nsd	–					
SK	nsd	nsd	nsd	–				
SMM	nsd	nsd	nsd	nsd	–			
TS	**	nsd	nsd	nsd	nsd	–		
TL	nsd	nsd	nsd	nsd	nsd	nsd	–	
WSK	nsd	nsd	**	nsd	nsd	*	nsd	–

DP, Dorper; EF, East Friesian; HS, Hu sheep; SK, Suffolk; SMM, South African Mutton Merino; TS, Tan sheep; TL, Texel; WSK, White Suffolk; nsd, no significant difference; * $P < 0.05$; ** $P < 0.01$.

Supplementary Figures

```
1                                                       acatggggatctgtctgtgttctgagacatggggagaaccagcatgtcagcca
54      acccagcagggtcttcacccagaccctttgatgcaggctttgcaaatcaacagggagaaagtccatgtggctacctgtgcttaatgcaga
144     gcaaaggaaaaactgaattgtatttgtgggcttcaaattgaggtcccatcagaatctgtctgtctgaacgtgaacatttgggtgattttg
234     atgtggcccaaactcccacaacgaatgtaccttcaggactcgttccggagtaggttcacgacgggcatcggggccccacccagactcctg
        M  W  P  K  L  P  Q  R  M  Y  L  Q  D  S  F  R  S  R  F  T  T  G  I  G  A  P  P  R  L  L
324     gagctggctggccagagcctgctgcagaatgaggccttggccatcatggctctggaggagctgcccattgaactcttcccaccgctgttt
        E  L  A  G  Q  S  L  L  Q  N  E  A  L  A  I  M  A  L  E  E  L  P  I  E  L  F  P  P  L  F
414     atggcggcctttgctgggaggcacaccaaggccctgaaggcgatggtgcaggcctggcccttcccctgcctcccactggggggccctgatg
        M  A  A  F  A  G  R  H  T  K  A  L  K  A  M  V  Q  A  W  P  F  P  C  L  P  L  G  A  L  M
504     aaggaccacccgcctcatctggagacatttcaggctgcactcgacggcctggacatcctgctttctcaggaggtccgcccccaggcggtgg
        K  D  H  P  P  H  L  E  T  F  Q  A  A  L  D  G  L  D  I  L  L  S  Q  E  V  R  P  R  R  W
594     aagctgcaggtgctggacttacgccggaacacccatgaggacttctggaccctgtggtctggcatcaaggctagcgtttgctcactgctg
        K  L  Q  V  L  D  L  R  R  N  T  H  E  D  F  W  T  L  W  S  G  I  K  A  S  V  C  S  L  L
684     gagcctgacccgatccagcccatgcagaagagccaaagggtggagtcacaggcagtactaaagctggtgcgggcccctgtggaggtgctg
        E  P  D  P  I  Q  P  M  Q  K  S  Q  R  V  E  S  Q  A  V  L  K  L  V  R  A  P  V  E  V  L
774     gtcgacctgtgcctcaaggaggacaccctggatgagacactcagctacctgctgaagaaggccaaacagaggaggagcctgctgcacctg
        V  D  L  C  L  K  E  D  T  L  D  E  T  L  S  Y  L  L  K  K  A  K  Q  R  R  S  L  L  H  L
864     cgctgccagaagctgaggatcttcaccatgcctatgcagagcatcaagaggatcctgaaggtggtgcagctggactccatccaggacctg
        R  C  Q  K  L  R  I  F  T  M  P  M  Q  S  I  K  R  I  L  K  V  V  Q  L  D  S  I  Q  D  L
954     gaggtgaactgcatctggaagctgggcacactgggcaggtttgtgccgcatctgggacggatgggaaatctgcgccggctgctgctgtca
        E  V  N  C  I  W  K  L  G  T  L  G  R  F  V  P  H  L  G  R  M  G  N  L  R  R  L  L  L  S
1044    cgcatccacatgttgccgcgcaccaccccggaccaggaggagcacttagtcgaccagctcaccacccagttcctgaacctgccccacctg
        R  I  H  M  L  P  R  T  T  P  D  Q  E  E  H  L  V  D  Q  L  T  T  Q  F  L  N  L  P  H  L
1134    caggagctctacctagactccatctccttcctcgagggcgcctgcaccaggtgctcaggtgcctgaagacccctctggagaccttgtgg
        Q  E  L  Y  L  D  S  I  S  F  L  E  G  R  L  H  Q  V  L  R  C  L  K  T  P  L  E  T  L  W
1224    atcaccaactgcctgctttcagagtcagacctgatgtacctgtcgcagtgcccgagcatcagcatgctgaaggacctgagccttagtggg
        I  T  N  C  L  L  S  E  S  D  L  M  Y  L  S  Q  C  P  S  I  S  M  L  K  D  L  S  L  S  G
1314    gtcaacctgaccagcctcagcttggaacctctgcaggtcctgatcgagtggacctcggccaccctacaggacctagacctaaatgagtgc
        V  N  L  T  S  L  S  L  E  P  L  Q  V  L  I  E  W  T  S  A  T  L  Q  D  L  D  L  N  E  C
1404    ggcatcatggacacccagttcagtgccctcctgccttccctgagttgctgctcccagctcactaccttcagcttctgtggcaaccccatc
        G  I  M  D  T  Q  F  S  A  L  L  P  S  L  S  C  C  S  Q  L  T  T  F  S  F  C  G  N  P  I
1494    tccatggccgtgctggagagcctacttcaacacaccatgggctgagcaagctaagccacgtgctgtaccccgcacccctggagagctat
        S  M  A  V  L  E  S  L  L  Q  H  T  M  G  L  S  K  L  S  H  V  L  Y  P  A  P  L  E  S  Y
1584    gaggatgtgaatggcactctgcacctgggcctcctggctgagcttcacacccggctcaagcagctgctgtataaatctgggagggccagc
        E  D  V  N  G  T  L  H  L  G  L  L  A  E  L  H  T  R  L  K  Q  L  L  Y  K  S  G  R  A  S
1674    atgggctgggtcagcgccagcccctgtccacactgcggtgaccagatcttctatgacactgagcccatcctgtgcccctgctacatgcca
        M  G  W  V  S  A  S  P  C  P  H  C  G  D  Q  I  F  Y  D  T  E  P  I  L  C  P  C  Y  M  P
1764    gcctagcccagctcacgagttgcaggctttgtcccccgcactgggacactgccacctagaccttacttcatctagaaggaactcaaacca
        A  *
1854    tggtttcagacaatagttcactgcatgctggcaaaggaaaggtgatttaaagtgggcatgggggagggcttggtgggattttggagag
1944    atgtgttcctacaaagttagaaatttgaatctgaatttggagggggaattttgggcttgggaggttgatggagtcgttacccctgtatt
2034    ggattgttacaaataaataaagggaacttcagtgtcaaaaaaaaaaaaaaaaaaaaaaaaaaa
```

Supplementary Figure S1. The complete cDNA nucleotide sequence and deduced amino acid sequence of ovine *PRAMEY*.

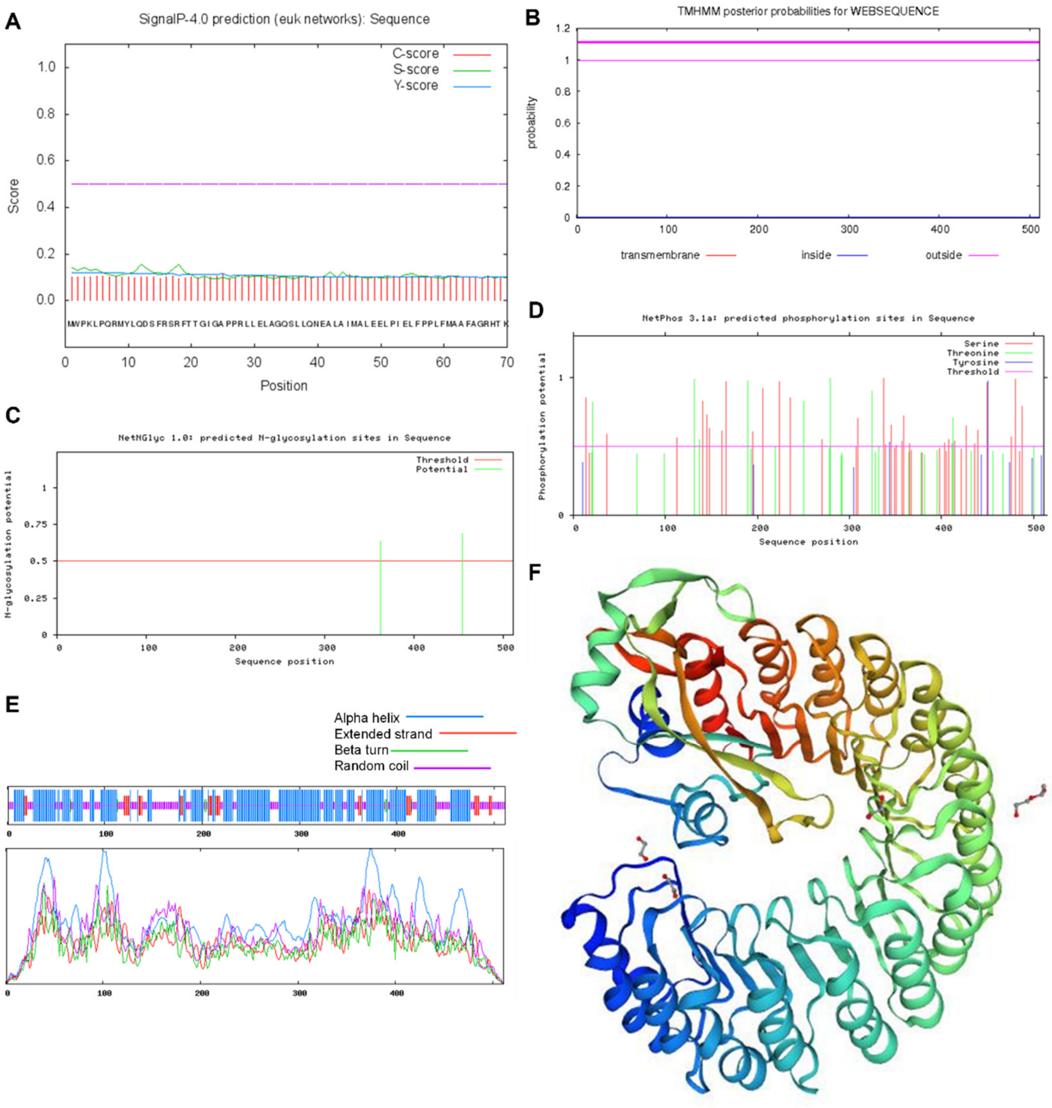

Supplementary Figure S2. The bioinformatics analysis of the predicted *PRAMEY* protein. (A) *PRAMEY* had no signal peptide site. (B) *PRAMEY* had no predictable transmembrane region. (C) *PRAMEY* protein had 2 N-glycosylation sites. (D) *PRAMEY* protein also had 31 N-glycosylation sites. (E) Secondary structures of ovine *PRAMEY* protein. (F) Tertiary structures of ovine *PRAMEY* protein.

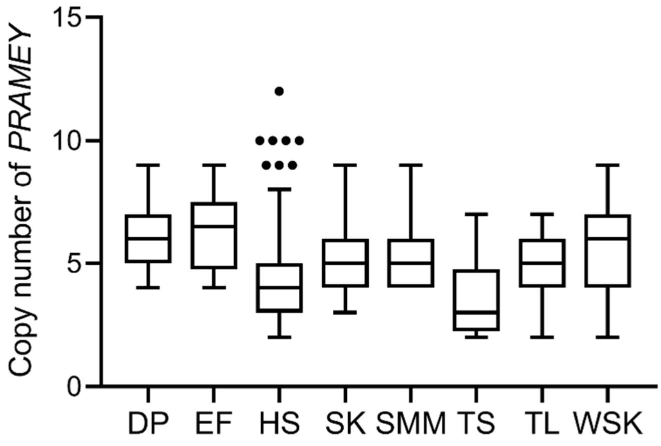

Supplementary Figure S3. Boxplot analysis of copy number of the *PRAMEY* among eight sheep breeds. The eight outliers in Hu sheep population are indicated by a solid circle. The x-axis represents the different sheep breeds. DP, Dorper; EF, East Friesian; HS, Hu sheep; SK, Suffolk; SMM, South African Mutton Merino; TS, Tan sheep; TL, Texel; WSK, White Suffolk.

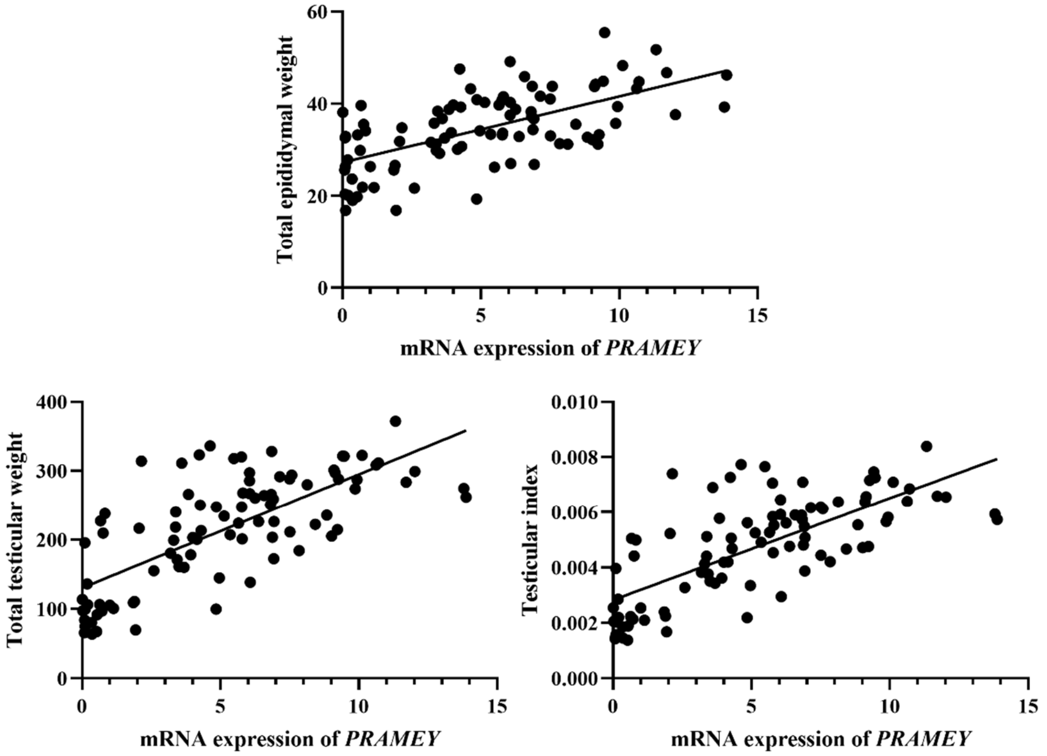

Supplementary Figure S4. Scatter diagram reflecting the correlations between mRNA expression of *PRAMEY* and testicular parameters. The *PRAMEY* expression was positively associated with total epididymal weight (TEW: r = 0.865, $P < 0.0001$), total testicular weight (TTW: r = 0.766, $P < 0.0001$), and testicular index (TI: r = 0.676, $P < 0.0001$). Each black dot represents one individual.
